# Novel Bio-functional Magnesium Coating on Porous Ti6Al4V Orthopaedic Implants: *In vitro* and *In vivo* Study

**DOI:** 10.1038/srep40755

**Published:** 2017-01-19

**Authors:** Xiaokang Li, Peng Gao, Peng Wan, Yifeng Pei, Lei Shi, Bo Fan, Chao Shen, Xin Xiao, Ke Yang, Zheng Guo

**Affiliations:** 1 Department of Orthopaedics, Xijing Hospital, Fourth Military Medical University, Xi’an, 710032, China; 2 Institute of Metal Research, Chinese Academy of Science, Shenyang 110016, China

## Abstract

Titanium and its alloys with various porous structures are one of the most important metals used in orthopaedic implants due to favourable properties as replacement for hard tissues. However, surface modification is critical to improve the osteointegration of titanium and its alloys. In this study, a bioactive magnesium coating was successfully fabricated on porous Ti6Al4V by means of arc ion plating, which was proved with fine grain size and high film/substrate adhesion. The surface composition and morphology were characterized by X-ray diffraction and SEM equipped with energy dispersive spectroscopy. Furthermore, the *in vitro* study of cytotoxicity and proliferation of MC3T3-E1 cells showed that magnesium coated porous Ti6Al4V had suitable degradation and biocompatibility. Moreover, the *in vivo* studies including fluorescent labelling, micro-computed tomography analysis scan and Van-Gieson staining of histological sections indicated that magnesium coated porous Ti6Al4V could significantly promote bone regeneration in rabbit femoral condylar defects after implantation for 4 and 8 weeks, and has better osteogenesis and osteointegration than the bare porous Ti6Al4V. Therefore, it is expected that this bioactive magnesium coating on porous Ti6Al4V scaffolds with improved osteointegration and osteogenesis functions can be used for orthopedic applications.

Titanium (Ti) and its alloys are one of the most important metals used in orthopaedic implants due to favourable properties of high strength, rigidity, fracture toughness and their reliable mechanical performance as replacement for hard tissues^1^,^2^,^3^. Now main clinical applications of titanium implants in orthopaedics include artificial joints, spinal fusion instruments, and fracture fixations such as plates, screws and intramedullary rods^4^,^5^,^6^,^7^. Although titanium based medical devices have been used clinically for more than 30 years, there are still weaknesses for the implants that need to be resolved. The lack of osteo-conduction and integration into the bone for long-term survival often occur and lead to implant failure^8^,^9^,^10^,^11^. Therefore the challenges for Ti-based implants are incorporating with osteo-integration, and also enhanced bioactivity with bone healing and regeneration, thus improving implant-host interactions so as to reduce biological related implant failure.

Many approaches for improving the bioactivity of Ti and its alloys have been studied. These surface modifications can be concluded into two kinds: (1) bioactive coatings, such as calcium phosphate, that accelerate bone formation^12^,^13^,^14^,^15^, and (2) physicochemical changes on the surface of metallic implants, such as the roughness and wettability, which could induce a firm bonding of the implants to bone^16^,^17^,^18^,^19^,^20^. Moreover, porous structure fabricated by three-dimension printing can also increase the ingrowth of bone and the anchorage of the implants^21^,^22^,^23^,^24^.

Recently amounts of studies on magnesium-based metals are conducted for their potential to be used as biodegradable implants due to their biocompatibility combined with good physical and mechanical properties^25^,^26^,^27^,^28^. Importantly, it was found that magnesium could influence bone tissue growth positively, which could improve the bone healing and reconstruction^29^,^30^. Witte *et al*. reported that magnesium-based bone implants showed effects on the surrounding bone tissues after implantation and high magnesium ion concentration could lead to bone cell activation^31^,^32^. Zhai *et al*. found that the degradation products of magnesium could also influence the proliferation and apoptosis of osteoblast and osteoclast^33^. Furthermore, magnesium exhibited antibacterial properties against Staphylococcus aureus that prevented bacterial attachment and formation of biofilm^34^,^35^,^36^. Therefore, biodegradable magnesium employed as a coating could be appropriate for medical implants, which is expected to promote drug-enhancing osteo-integration and reduce implant infection compared with conventional metals and coating used today.

In this work, magnesium was fabricated as coating instead of substrate for the first time. The biofunctional magnesium coating was fabricated by arc ion plating, which was proved with fine grain size and high film/substrate adhesion in comparison with other PVD methods^37^. Microstructure, morphology and composition of the magnesium coating were investigated by means of SEM, EDS and XRD. *In vitro* degradation and ions releasing were measured after immersion in simulated body fluids. Furthermore, cytocompatibility and animal implantation tests were done to evaluate the related cell attachment, viability and bone response *in vivo.*

## Materials and Methods

### Materials preparation

**Figure 1:**
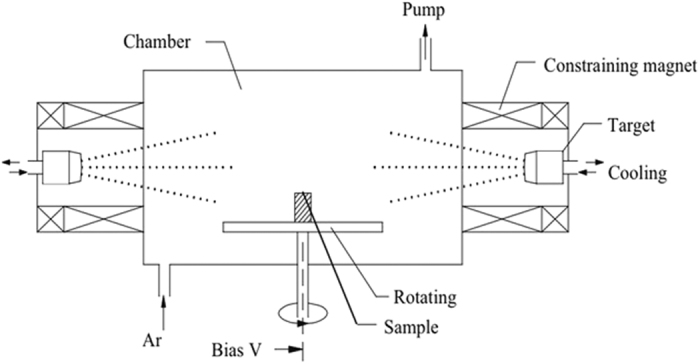
Schematic diagram of pulse biased arc ion plating system.

The deposition of magnesium coating on Ti6Al4V alloy was processed with a Bulat 6 arc ion plating system^38^, as shown in Fig. 1. The Ti6Al4V samples with dimensions of Φ15 × 2 mm^3^ and 3D printed porous cylinder with dimensions of Φ 2 × 12 mm^3^ and Φ 6 × 8 mm^3^ were used as substrate and subsequently for *in vitro* evaluation and *in vivo* implantation, respectively. All the samples were polished and rinsed with acetone in an ultrasonic bath for 20 min. A high purity Mg (99.99%) target was used to bombard and sputter the substrate surface with a constant target arc current of 50 A, P_Ar_ = 3.5 × 10^−2^ Pa, for 5 min. The current density used in the negative bias voltage application was in the range of 0.12~0.16 A. During deposition, a pulsed power source superimposed a negative pulse bias to the substrates with the following parameters: pulse bias magnitude U_p_ = 100 V, pulse frequency f = 30 kHz, and duty ratio D = 40%; and the following parameters were maintained constant: two arc source currents I_Mg_1__ = I_Mg_2__ = 0.1 A, P_Ar_ = 3.5 × 10^−2^ Pa, the distance between samples and cathode arc targets 400 mm, and the total deposition time 60 min. During deposition, substrate temperature T_s_ was approximately 245 °C.

### Characterization of the coating

Structural characterization of the deposited films was carried out by X-ray diffraction (XRD, Rigaku D/Max 2500PC, Tokyo, Japan) with Cu-K_α_ radiation. The XRD pattern was made with MDI Jade 5.0 software (Materials Data Inc., CA, USA). The surface morphology and composition were examined by scanning electron microscopy (SEM, HITACHI S-3400N, Japan) equipped with energy dispersive spectroscopy (EDS, Oxford INCA energy 300).

### *In vitro* degradation tests

The samples were immersed in Hank’s solution (8.00 g/l NaCl, 0.40 g/l KCl, 0.12 g/l Na_2_HPO_4_, 0.06 g/l KH_2_PO_4_, 0.14 g/l CaCl_2_, 0.20 g/l MgSO_4_, 0.35 g/l NaHCO_3_ and 1.00 g/l glucose) for 7 days at 37 ± 0.5 °C with the immersion ratio of 1.25 cm^2^/ml and 0.2 g/ml for 3D printing porous cylinder according to ISO 10993-12. The immersion solutions were refreshed everyday to simulate the *in vivo* condition. The pH value of the solutions was recorded during the immersion process at intervals. Besides, the total release of Mg ions in the extracts were estimated using atomic absorption spectrophotometer (AAS, Hitachi Z2000, Japan) with the Hank’s solution as a medium control. All the tests were performed in triplicate.

### Cell culture

Mouse preosteoblast cells (MC3T3-E1) were gifts offered by the Center Laboratory for Tissue Engineering, College of Stomatology, Fourth Military Medical University, Xi’an, China^39^. The MC3T3-E1 cells were cultured in a condition of 5% CO_2_ and 37 °C using α-MEM complete medium supplemented with 10% heat-inactivated fetal bovine serum, 100 mg/ml streptomycin and 100 U/ml penicillin, as described in our previous study^40^. The medium was changed every other day thereafter.

### Cytotoxicity and cell proliferation experiments

Cellcytotoxicity and proliferation were evaluated using cell counting kit-8(CCK-8, Dojindo, Japan). The MC3T3-E1 cells were incubated and seeded on the samples (magnesium porous Ti6Al4V with and without magnesium coating) in 24-well plates at a density of 4 × 104 cells per well in the previously described medium. The samples were cultured for 1, 4, and 7 days. The culture medium was used as negative control. After cultivation to each time point, all the samples digested with trypsin-EDTA solution were then transferred to new 24-well culture plates. A 10% volume of the medium CCK-8 solution was then added to the wells and incubated at 37 °C for 2 h. After the reaction, 100 μL of the reaction solution was transferred to a new 96-well plate, and the optical density was measured at 450 nm by a microplate reader^41^. All the experiments were performed in duplicate wells and repeated three times.

### Animals and surgical procedures

For *in vivo* experiments, bare porous Ti6Al4V (group A) and magnesium coated porous Ti6Al4V (group B) samples were implanted into the lateral femoral epicondyle of male New Zealand white rabbits. Twenty-four rabbits with an average weight of 3 ± 0.5 kg were randomly divided into two groups according to the different implanting materials (n = 12 in each group). The surgical procedures were performed as described previously^40^. The rabbits were narcotized with 0.5 mg·kg^−1^ acepromazine and 10 mg·kg^−1^ ketamine via intravenous injection. The surgical areas were shaved and sterilized, and an incision about 1 cm long was made to expose the lateral femoral epicondyle. After exposed the lateral femoral epicondyle, a cylindrical defect (6 mm in diameter and 8 mm in length) was drilled. Then the scaffold materials were randomly inserted into the defects. After sufficient irrigation with normal saline, the wound was closed layer by layer. Antibiotics were intramuscularly injected postoperatively twice for 3 days to prevent wound infection. The rabbits were sacrificed by intravenous injection with an overdose of anaesthetic at 4 and 8 weeks post-operation, and the implants were harvested and soaked in 75% ethanol for further analysis.

The surgery and treatment of rabbits were performed strictly according to the regulations and laws of the Standing Committee on Ethics in China. The animal experiments were conducted at the Laboratory for Animal Research of the Research Institute of Orthopaedics at Xi’Jing Hospital affiliated with the Fourth Military Medical University in China and were approved by the Fourth Military Medical University Committee on Animal Care.

### Fluorochrome labelling

Sequential fluorochrome markers were administered to monitor the mineralization process of new bone formation. At 2 weeks and 3 days prior to sacrifice day, the animals were injected with tetracycline (50 mg/kg) and calcein (25 mg/kg), respectively. After the animals were sacrificed by intravenous injection with an overdose of anaesthetic, samples were obtained and fixed in 75% ethanol for two weeks before fluorescence analysis. This method was described in our previous work^41^.

### Micro-computed tomography (Micro-CT) evaluation

In order to evaluate the new bone formation around the implants, all the samples (n = 12 in each group) were fixed in 75% ethanol for two weeks, and then scanned by Micron X-ray 3D Imaging System (Y. Cheetah, Germany). The X-ray source voltage was set at 90 kV, and the beam current was 50.0 μA. The area of the samples was selected as the region of interest (ROI). The 450 projections were reconstructed using a modified parallel Feldkampalgorithm, and segmented into binary images (12-bit TIF images). The percentage of new bone volume out of ROI (BV/TV) was calculated using VG Studio MAX software with beam hardening correction which can decrease metal artifacts in micro-CT results. The thresholds were set at 200–1399 for new bone and 1400–4100 for implants, respectively.

### Histological examination and quantitative histological analysis

After micro-CT evaluation, the samples were dehydrated in a graded series of ethanol (80–100%) and cleared with toluene, and then embedded in methylmethacrylate to polymerize. After polymerization, a Leica cutting and grinding system (Leica Microtome, Wetzlar, Germany) was used to obtain approximately 70-mm-thick serial transverse sections. Before histological staining, the sections were observed under a fluorescence microscope (Penguin 600CL, Pixera). Then, the sections were stained with 1.2% trinitrophenol and 1% acid fuchsin (Van Gieson staining) and examined under a standard light microscope (Leica LA Microsystems, Bensheim, Germany) equipped with a digital image capture system (Penguin 600CL, Pixera). Bone and scaffold materials were measured with the help of a digital image analysis system (Image-ProPlus software, Silver Spring, USA). Bone and scaffold material volumes were calculated and compared statistically based on Van Gieson staining.

### Statistical analysis

The quantitative results were presented as the means ± SEM for each group. A one-way ANOVA test was used to perform the statistical analysis among different groups using PASW Statistics 19.0 software (SPSS Inc., Chicago, USA). *P* < 0.05 was considered statistically significant. GraphPad Prism 6.0 software (GraphPad Software Inc., La Jolla, USA) was used to plot graphs.

## Results and Discussions

### Characterization of magnesium coating

**Figure 2:**
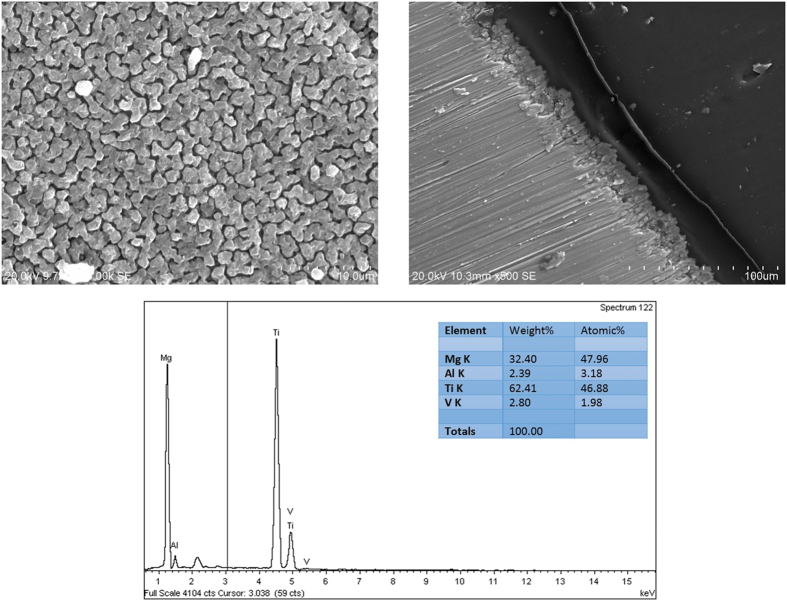
Surface, cross-sectional morphologies and EDS analysis of the magnesium coating on Ti6Al4V substrate.

**Figure 3:**
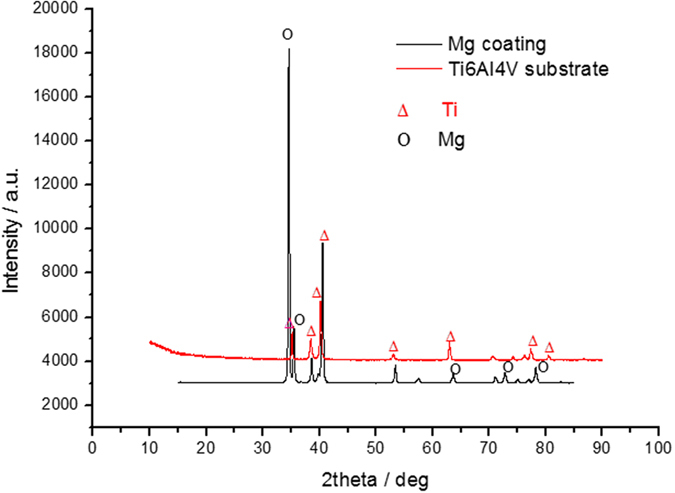
XRD pattern of the magnesium coated Ti6Al4V.

Figure 2 shows the surface and cross-sectional morphologies of the coating obtained by arc ion plating on Ti6Al4V substrate. The deposited film grew more densely with a smoother surface and no defect was observed. The coating consisted of uniform Mg grains with size of about 1 μm. It was reported that the grain size was controlled by the deposition pressure of the total reactor^42^. At a low pressure (P_Ar_ = 10^−2^ MPa) it would yield a higher nucleation rate, which resulted in a higher number of grains but of a smaller size. The thickness of the coating was approximately 5 μm. According to the EDS and XRD results (Fig. 3), pure magnesium was observed on the surface of Ti6Al4V substrate.

### *In vitro* degradation

**Figure 4:**
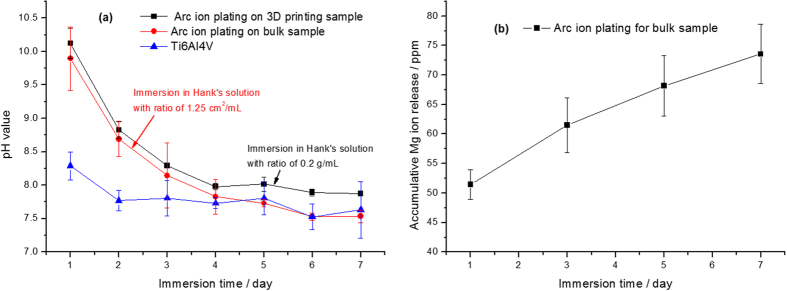
pH monitoring and ions releasing of the magnesium coated Ti6Al4V immersed in Hank’s solution for 7 days.

The variations of pH value of magnesium coatings on bulk and 3D printing porous samples compared with Ti6Al4V substrate during immersion periods in Hank’s solution are presented in Fig. 4(a). The pH value after 1 day immersion quickly reached to about 10, which was due to the accumulation of OH^−^ ions when the Mg(OH)_2_ was produced on surfaces of the samples. Then the trends of the pH values of the coatings were all on a gradual descending with extending the immersion time to 7 days. Severe degradation led to more consumption of magnesium on the surface of samples, which resulted in the decrease of the pH values. Whereas, the pH value of Ti6Al4V substrate always kept a low level of approximately 8. There was no significant discrepancy between the pH variation of magnesium coating on the bulk sample and 3D printing porous sample. Moreover, the magnesium ions released from the coating during different immersion periods showed a rising tendency, and with the immersion time the accumulation of magnesium ions releasing gradually increased to about 73 ppm after 7 days immersion, indicating a continuous releasing due to the degradation of magnesium.

### *In vitro* cytotoxicity and cell proliferation

**Figure 5:**
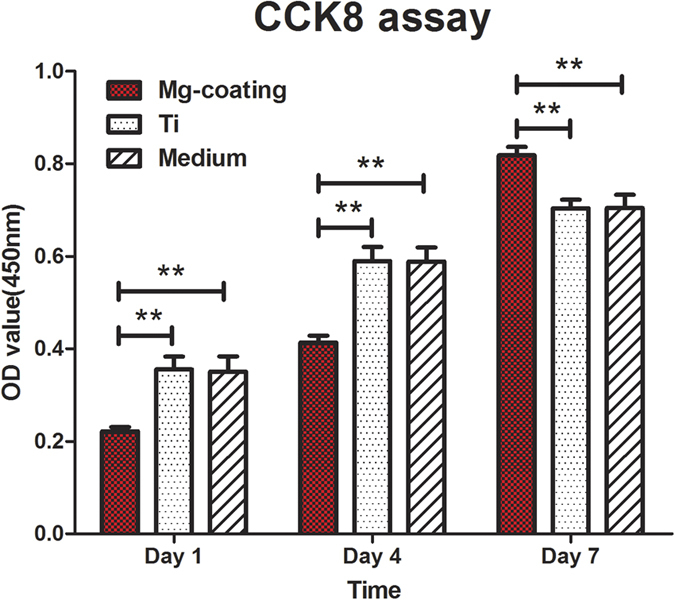
Cell cytotoxicity and proliferation co-cultured with Ti6Al4V control and Mg coating group for 1, 4, 7 days by CCK-8 test. For each group, n = 3; asterisks (**) indicate statistical significance, *P* < 0.05.

MC3T3-E1 cell cytotoxicity and proliferation were evaluated by the CCK-8 assay. After incubation with Mg coated porous Ti6Al4V and bare porous Ti6Al4V, as well as sole culture medium for 1 day and 4 days, cell proliferation showed lower on the Mg coated porous Ti6Al4V than the bare porous Ti6Al4V and sole culture medium (both *P* < 0.05). However, after incubation for 7 days, cell proliferation on samples co-cultured with Mg coated porous Ti6Al4V was much higher than those with bare porous Ti6Al4V and sole culture medium (both *P* < 0.05), as shown in Fig. 5. The bare porous Ti6Al4V and sole culture medium have no obvious difference on MC3T3-E1 cell cytotoxicity and proliferation at each time point (both *P* > 0.05). These results indicate that the Mg coated porous Ti6Al4V might suppress the MC3T3-E1 cell proliferation before day 4 compared with bare porous Ti6Al4V, nevertheless, it could improve the MC3T3-E1 cell proliferation after day 4.

### Fluorochrome labelling

**Figure 6:**
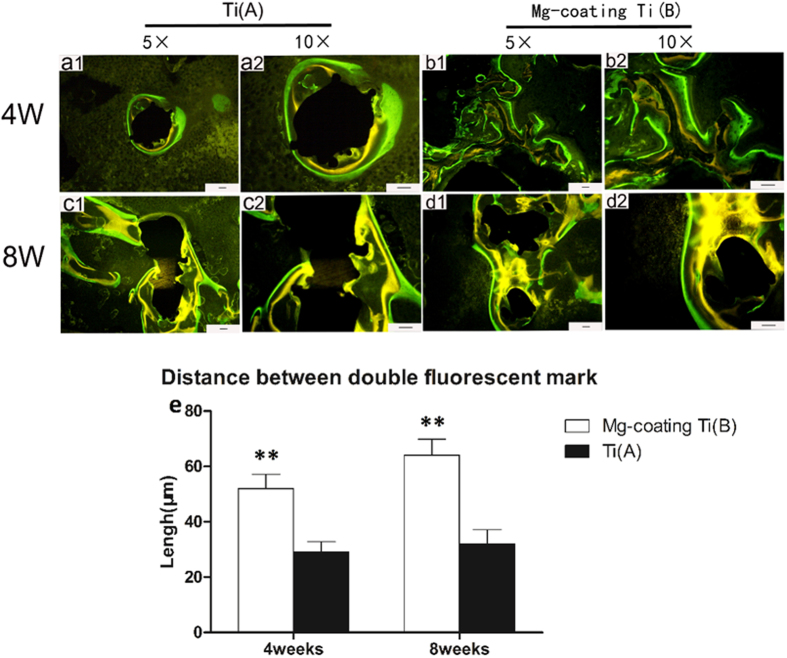
Fluorochrome labelling of regenerated bone in different implants at 4 weeks (**a1**, **a2**, **b1** and **b2**) and 8 weeks (**c1**, **c2**, **d1** and **d2**) post-operations in bare porous Ti6Al4V group (**a1**, **a2**, **c1** and **c2**) and Mg coated porous Ti6Al4Vgroup (**b1**, **b2**, **d1** and **d2**). (**e**) Quantitative analysis results at different time points post-operation. Scale bar: 50 μm (black) (asterisks (**) indicate statistical significance; *P* < 0.05).

Fluorescent labelling was evaluated on Mg coated porous Ti6Al4V and bare porous Ti6Al4V, as shown in Fig. 6. Yellow lines indicate the newly deposited calcification with calcein, whereas the green lines indicate the deposition of new calcification with tetracycline. The interval between the two lines represents the rate of newly deposited calcification, which indicates the new bone formation rate. Quantitative analysis of the fluorochrome marker intervals revealed that calcification deposition was significantly higher in Mg coated porous Ti6Al4V than that in bare porous Ti6Al4V at both 4 weeks (Fig. 6a1 and b2) and 8 weeks (Fig. 6c1) post-operations. At 4 weeks, the calcification deposition on Mg coated porous Ti6Al4V was higher than that on bare porous Ti6Al4V. Moreover, after implantation for 8 weeks, the difference was much higher than 4 weeks. Furthermore, both groups exhibited increased calcification deposition throughout the study.

### Micro-CT evaluation

**Figure 7:**
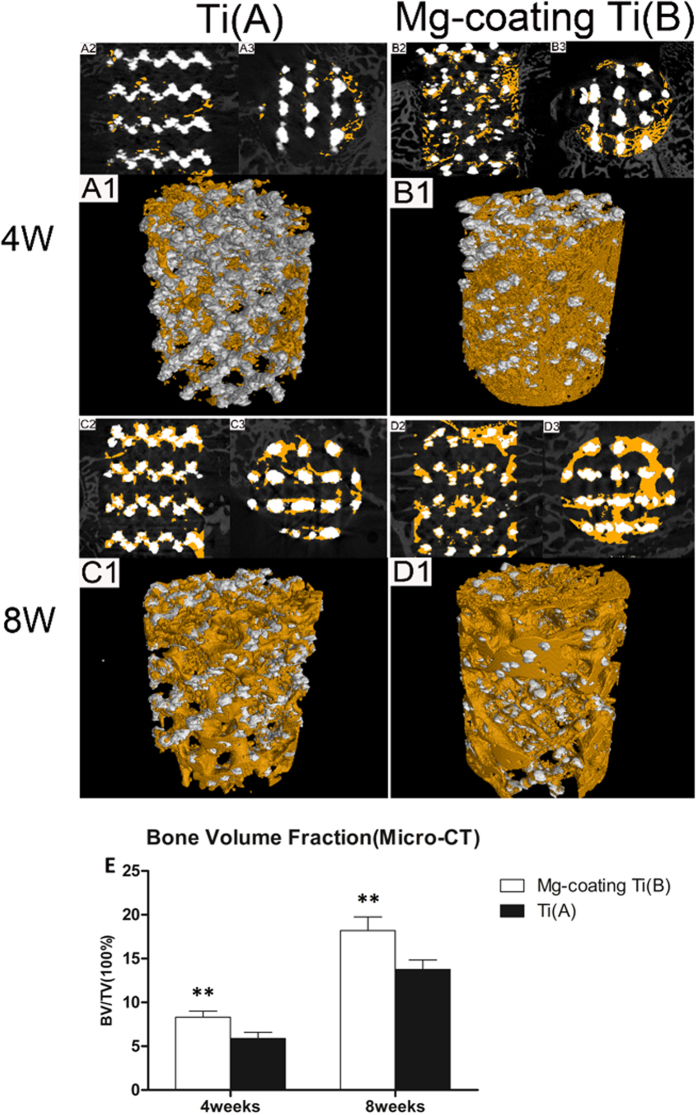
Micro-CT images of the bare porous Ti6Al4V (group A) and Mg coated porous Ti6Al4V (group B) at 4 weeks and 8 weeks after implantation. The yellow colour component was newly formed bone in these scaffolds. (**E**) Percentages of regenerated bone volume/total volume (BV/TV) in these implants. Asterisks (**) indicated statistical significance compared to the group A, *P* < 0.05.

Typically reconstructed 3D stereoscopic pictures of scaffolds and the growth of newly formed bone into scaffolds were obtained by Micro-CT scan. The new bone ingrowth was evaluated at 4 weeks and 8 weeks after implantation (Fig. 7). The cross sections and the 3D reconstruction images are shown in Fig. 7A2 and A–D. The newly formed bone tissue was observed to integrate into two kinds of scaffolds implants, and the values of regenerated bone volume/total volume (BV/TV) increased during the study of both bare porous Ti6Al4V and Mg coated porous Ti6Al4V. Additionally, quantitative volumetric analysis revealed that the BV/TV of Mg coated porous Ti6Al4V (group A) (Fig. 7A1–A3) was higher than that of bare porous Ti6Al4V (group B) (Fig. 7B1–B3) at 4 weeks after implantation (*P* < 0.05). After implantation for 8 weeks, the new bone formation was significantly higher in group A than that in group B, and the difference was statically significant (*P* < 0.05).

### Histological examination of new bone formation

No gas was found near the soft tissue around the defect and scaffolds. Van Gieson staining was performed in histological analysis to assess the osteogenesis and osteointegration of the bare porous Ti6Al4V (group A) and Mg coated porous Ti6Al4V (group B) at 2, 4 and 8 weeks after implantation. The quantitative analysis results showed that trabeculae of the regenerated bone grew into the scaffolds in both bare porous Ti6Al4V (group A) and Mg coated porous Ti6Al4V (group B). At 2 weeks after implantation, a small amount of new bone was observed near the edge of the scaffolds and abundant connective tissue was found to be filled in the center of the scaffolds in both groups. There was no significantly difference between these two groups (*P* > 0.05). After implantation for 4 weeks, more trabeculae of the regenerated bone grew into the scaffolds in both groups, and group B have more regenerated bone than group A (*P* < 0.05). At 8 weeks, the new bone increased and connective tissue decreased in both groups. Moreover, newly formed bone in group B was much more than that in group A (*P* < 0.05). Notably, newly formed bone was observed to grow deep into the scaffold in Mg coated porous Ti6Al4V while newly formed bone was confined near the edge of the scaffold and few grew deep into the scaffold in bare porous Ti6Al4V (Fig. 7E1–F3). The above results revealed that the Mg coating substantially improved the osteogenesis and osteointegration properties of porous Ti6Al4V scaffolds.

## Discussion

The porous titanium and its alloy with controllable geometry, pore size and porosity are the materials of choice for mostendosseous implants in dental and orthopaedic bone defect clinical uses^43^,^44^,^45^,^46^. Bone healing and growing into titanium implant is recognized to follow a sequential and overlapping series of events including osteoblastic lineage attachment and cell proliferation, differentiation which eventually lead to bone tissue regeneration^47^. Nevertheless, how to improve the osteointegration and osteogenesis properties of these porous titanium and its alloy is still a major concern for their clinical applications^8^,^9^,^10^,^11^. Magnesium and its alloys have been considered as a revolutionary biodegradable implant material and are widely studied recently as a new type of biomedical materials due to the prominent advantages in mechanical property, biodegradability and biocompatibility^48^,^49^,^50^. However, the major drawbacks which limit the clinical use of magnesium and its alloys are fast initial degradation rate and low mechanical strength support which mismatch with the new bone formation in bone defect^48^.

In this study, we combined the advantages of both magnesium and titanium and its alloy, trying to limit the disadvantages as much as possible. Instead of using as substrate, magnesium was fabricated as the coating on porous titanium alloy (Ti6Al4V) scaffold surfaces through arc ion plating method, which was proved with fine grain size and high film/substrate adhesion. A high purity Mg (99.99%) target was used to bombard and sputter the substrate surface with a constant target arc current of 50 A, PAr = 3.5 × 10^−2^ Pa for 5 min. The current density was in the range of 0.12~0.16 A. During deposition, a pulsed power source superimposed a negative pulse bias to the substrates with pulse bias magnitude Up = 100 V, pulse frequency f = 30 kHz, and duty ratio D = 40%. Two arc source currents I Mg1 = I Mg2 = 0.1 A, PAr = 3.5 × 10^−2^ Pa. The distance between samples and cathode arc targets 400 mm, and the total deposition time 60 min. During deposition, substrate temperature Ts was approximately 245 °C. Our results revealed that the resulting magnesium coating enhanced proliferation of MC3T3-E1 cells on the Mg coated porous Ti6Al4V scaffolds *in vitro* and improved osteointegration of the scaffolds *in vivo* compared with bare porous Ti6Al4V scaffolds.

Previous studies showed that magnesium and its alloy could corrosion *in vitro* and increased the pH of the culture medium, which would be harmful to the cell survival^50^. Cytotoxicity test serves as an important indicator for quickly detecting the biocompatibility of Mg coated porous Ti6Al4V scaffold. In theory, no metals have an unlimited intake in the human body. Many alloying elements may cause toxic reactions beyond the tolerance limit^51^,^52^. The biocompatibility of developed Mg coated porous Ti6Al4V scaffold is influenced by the amount of the released magnesium element, which is related to the corrosion rate of the Mg coated porous Ti6Al4V scaffold in the application environment^48^. In this study, *in vitro* degradation tests including cell cytotoxicity and proliferation were evaluated (Figs 4 and 5). The pH value after 1 day immersion quickly reached to about 10, which was due to the accumulation of OH^−^ ions from the Mg(OH)_2_ produced on the surfaces of the samples. After 4 days immersion, the pH value decreased to approximate 8 and became relatively stable subsequently. Whereas, the pH value of Ti6Al4V substrate always kept at a low level of approximately 8. These results revealed that the Mg coating might corrosion relatively rapid in the first 4 days after immersion but became stable and slow after 4 days of immersion. MC3T3-E1 cell cytotoxicity and proliferation were evaluated by the CCK-8 assay. After incubation for 1 day and 4 days, cell proliferation was lower on the Mg coated porous Ti6Al4V than the bare porous Ti6Al4V. However, the situation was reversed at 7 days cultivation. Cell proliferation on the Mg coated porous Ti6Al4V was much higher than that on the bare porous Ti6Al4V (Fig. 5). These results indicate that the Mg coated porous Ti6Al4V might suppress the MC3T3-E1 cell proliferation before 4 days compared with the bare porous Ti6Al4V. Nevertheless, Mg coated porous Ti6Al4V could improve MC3T3-E1 cell proliferation after 4 days.

Mg coating corrosion is the production of magnesium hydroxide and hydrogen gas. With exposure to high chloride concentrations such as in a physiological environment, Mg(OH)_2_ reacts with chloride ions to produce MgCl_2_, which is highly soluble^53^. This promotes the rapid dissolution of the Mg coating, with the subsequent production of hydrogen gas and hydroxide ions. The surface roughness of Mg coating influences the corrosion rate in the physiological environment. Meanwhile, the corrosion behavior change on different physiological environments such as solutions as well as anion types and concentrations^54^. Corrosion in pure water and in basic solutions is relatively slow. However, the corrosion rate must be considerable in a liquid chloride and in acid solutions.

**Figure 8:**
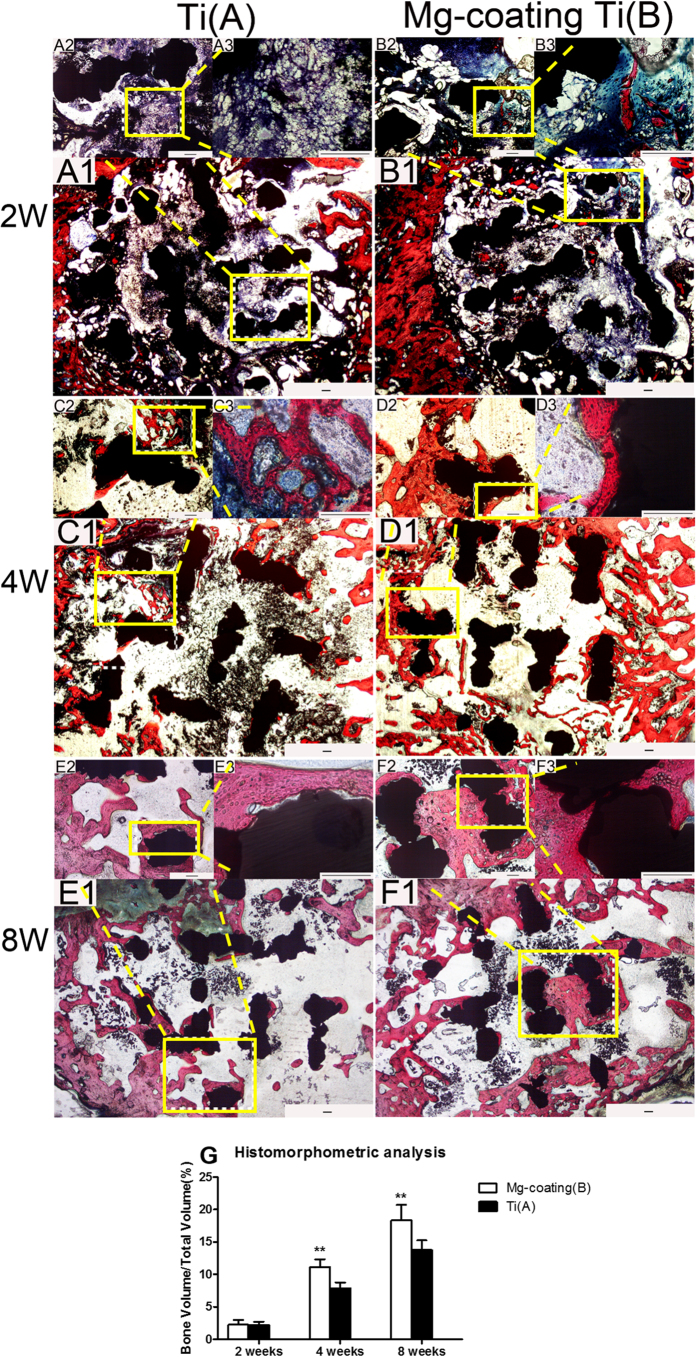
(**A1–F3**) Van-Gieson staining of histological sections and (**G**) histomorphometric analysis of the bare porous Ti6Al4V and Mg coated porous Ti6Al4V implants at 2, 4 and 8 weeks post operation. The tissue stained with red colour was the newly formed bone. Asterisks (**) indicate statistical significance compared to the bare porous Ti6Al4V, *P* < 0.05. Scale bar = 200 μm.

Apart from the *in vitro* evaluation about the scaffolds, the *in vivo* new bone regenerating ability of both scaffolds was detected. A defect model of rabbit femoral condylar was developed to investigate the osteogenesis and osteointegration properties of Mg coated porous Ti6Al4V and bare porous Ti6Al4V scaffolds *in vivo*. Fluorescent labelling results revealed that the porous Ti6Al4V coated with Mg had higher newly deposited calcification rate than the bare porous Ti6Al4V at both 4 weeks and 8 weeks post-operation (Fig. 6). Furthermore, both groups exhibited increased calcification deposition throughout the study. Typically reconstructed 3D stereoscopic pictures of newly formed bone growing into scaffolds were obtained by Micro-CT scan. The value of newly regenerated bone volume/total volume (BV/TV) on the Mg coated porous Ti6Al4V was higher than that on the bare porous Ti6Al4V at 4 weeks and 8 weeks after implantation (Fig. 7). Furthermore, Van Gieson staining was performed in histological analysis and revealed that trabeculae of the regenerated bone grew into the scaffolds and no gas was found near the soft tissue around the defect and scaffolds. More trabeculae of the regenerated bone and less connective tissue grew into the scaffolds throughout the study, and newly formed bone on the Mg coating porous Ti6Al4V was much higher and grew deeper into the scaffold than the bare porous Ti6Al4V after implantations for 4 weeks and 8 weeks (Fig. 8). The above results revealed that the Mg coating substantially improved the osteogenesis and osteointegration properties of porous Ti6Al4V scaffolds.

The degradation of Mg coating could release the magnesium ion in the implant area which recruit a sequential and overlapping series of events including osteoblastic lineage attachment and cell proliferation, differentiation which eventually lead to bone tissue regeneration. The magnesium ion released from the coating could transport to the periosteal region through Harversian’s or Volkmann’s canals since the diameter of magnesium ion (<300 pm) is much smaller than that of Harversian’s or Volkmann’s canals^55^. The bone cells were recruited to the surface and inside of the titanium alloy and facilitated the new bone formation.

Thus, in this work, we herein demonstrate that the Mg coated porous Ti6Al4V has suitable corrosion rate and biocompatibility *in vitro*, and has better osteogenesis and osteointegration properties than the bare porous Ti6Al4V *in vivo*. Further study of osteogenesis about Mg coated porous Ti6Al4V *in vitro* and longer time observation of new bone formation *in vivo* need to be investigated.

## Conclusion

In this study, bioactive Mg coating was successfully fabricated on porous Ti6Al4V by arc ion plating, which was proved with fine grain size and high film/substrate adhesion. Furthermore, the *in vitro* study showed that the Mg coated porous Ti6Al4V had suitable corrosion rate and biocompatibility. Moreover, *in vivo* studies including fluorescent labelling, Micro-CT scan and Van Gieson staining indicated that the Mg coated porous Ti6Al4V had better osteogenesis and osteointegration properties than the bare porous Ti6Al4V.

## Additional Information

**How to cite this article:** Li, X. *et al*. Novel Bio-functional Magnesium Coating on Porous Ti6Al4V Orthopaedic Implants: *In vitro* and *In vivo* Study. *Sci. Rep.* **7**, 40755; doi: 10.1038/srep40755 (2017).

**Publisher's note:** Springer Nature remains neutral with regard to jurisdictional claims in published maps and institutional affiliations.

## References

[b1] Clavell R. S., de, Llano J. J., Carda C., Ribelles J. L. & Vallés-Lluch A. *In vitro* assessment of the biological response of Ti6Al4V implants coated with hydroxyapatite microdomains. J Biomed Mater Res A. 104(11), 2723–9 (2016). 27341787 10.1002/jbm.a.35817

[b2] Diefenbeck M. . Gentamicin coating of plasma chemical oxidized titanium alloy prevents implant-related osteomyelitis in rats. Biomaterials. 101, 156–64 (2016). 27294535 10.1016/j.biomaterials.2016.05.039

[b3] Guillot R. . Assessment of a polyelectrolyte multilayer film coating loaded with BMP-2 on titanium and PEEK implants in the rabbit femoral condyle. Acta Biomater. 36, 310–22 (2016). 26965394 10.1016/j.actbio.2016.03.010 PMC5015710

[b4] Xu J. . Potential use of porous titanium-niobium alloy in orthopedic implants: preparation and experimental study of its biocompatibility *in vitro*. PLoS One. 8(11), e79289, doi: 10.1371/journal.pone.0079289. eCollection (2013). PMC3834032 24260188

[b5] Smith J. S. . Prospective multicenter assessment of risk factors for rod fracture following surgery for adult spinal deformity. J Neurosurg Spine. 21(6), 994–1003 (2014). 25325175 10.3171/2014.9.SPINE131176

[b6] Ahmad F. U., Sidani C., Fourzali R. & Wang M. Y. Postoperative magnetic resonance imaging artifact with cobalt-chromium versus titanium spinal instrumentation: presented at the 2013 Joint Spine Section Meeting. Clinical article. Clinical article. J Neurosurg Spine. 19(5), 629–36 (2013). 24053373 10.3171/2013.7.SPINE1359

[b7] Decker S. . A nickel-titanium shape memory alloy plate for contactless inverse dynamization after internal fixation in a sheep tibia fracture model: A pilot study. Technol Health Care. 23(4), 463–74 (2015). 26409909 10.3233/THC-150912

[b8] Xue W., Krishna B. V., Bandyopadhyay A. & Bose S. Processing and Biocompatibility Evaluation of Laser Processed Porous Titanium. Acta Biomater 3(6), 1007–18 (2007). 17627910 10.1016/j.actbio.2007.05.009

[b9] Murphy C. M., Haugh M. G. & O’Brien F. J. The effect of mean pore size on cell attachment, proliferation and migration in collagen-glycosaminoglycan scaffolds for bone tissue engineering. Biomaterials. 31(3), 461–6 (2010). 19819008 10.1016/j.biomaterials.2009.09.063

[b10] Minagar S., Wang J., Berndt C. C., Ivanova E. P. & Wen C. Cell response of anodized nanotubes on titanium and titanium alloys. J Biomed Mater Res A. 101(9), 2726–39 (2013). 23436766 10.1002/jbm.a.34575

[b11] Drago C. & Howell K. Concepts for designing and fabricating metal implant frameworks for hybrid implant prostheses. J Prosthodont. 21(5), 413–24 (2012). 22413997 10.1111/j.1532-849X.2012.00835.x

[b12] Rafieerad A. R., Ashra M. R., Mahmoodian R. & Bushroa A. R. Surface characterization and corrosion behavior of calcium phosphate-base composite layer on titanium and its alloys via plasma electrolytic oxidation: A review paper. Mater Sci Eng C Mater Biol Appl. 57, 397–413 (2015). 26354281 10.1016/j.msec.2015.07.058

[b13] Schickle K. . Preparation of spherical calcium phosphate granulates suitable for the biofunctionalization of active brazed titanium alloy coatings. Biomed Tech (Berl). 60(2), 105–14 (2015). 25389977 10.1515/bmt-2014-0017

[b14] Le V. Q. . Alternative technique for calcium phosphate coating on titanium alloy implants. Biomatter. 4, e28534, doi: 10.4161/biom.28534 (2014). 24646569 PMC4010538

[b15] Park K. D. . Effect of magnesium and calcium phosphate coatings on osteoblastic responses to the titanium surface. J Adv Prosthodont. 5(4), 402–8 (2013). 24353877 10.4047/jap.2013.5.4.402 PMC3865194

[b16] Migita S., Okuyama S. & Araki K. Sub-micrometer scale surface roughness of titanium reduces fibroblasts function. J Appl Biomater Funct Mater. 14(1), e65–9 (2016). 26689819 10.5301/jabfm.5000260

[b17] Elias C. N., Fernandes D. J., Resende C. R. & Roestel J. Mechanical properties, surface morphology and stability of a modified commercially pure high strength titanium alloy for dental implants. Dent Mater. 31(2), e1–e13, doi: 10.1016/j.dental.2014.10.002. (2015). 25458351

[b18] Salou L., Hoornaert A., Louarn G. & Layrolle P. Enhanced osseointegration of titanium implants with nanostructured surfaces: an experimental study in rabbits. Acta Biomater. 11, 494–502 (2015). 25449926 10.1016/j.actbio.2014.10.017

[b19] Zuldesmi M., Waki A., Kuroda K. & Okido M. Hydrothermal treatment of titanium alloys for the enhancement of osteoconductivity. Mater Sci Eng C Mater Biol Appl. 49, 430–5 (2015). 25686969 10.1016/j.msec.2015.01.031

[b20] Kim Y. S., Shin S. Y., Moon S. K. & Yang S. M. Surface properties correlated with the human gingival fibroblasts attachment on various materials for implant abutments: a multiple regression analysis. Acta Odontol Scand. 73(1), 38–47 (2015). 25183254 10.3109/00016357.2014.949845

[b21] Lee H. U. . Innovative three-dimensional (3D) eco-TiO_2_ photocatalysts for practical environmental and bio-medical applications. Sci Rep. 4, 6740, doi: 10.1038/srep06740 (2014). 25338845 PMC4206844

[b22] Xiu P. . Tailored surface treatment of 3D printed porous Ti6Al4V by micro-arc oxidation for enhanced osseointegration via optimized bone in-growth patterns and interlocked bone/implant interface. ACS Appl Mater Interfaces. 8(28), 17964–75 (2016). 27341499 10.1021/acsami.6b05893

[b23] Gagg G., Ghassemieh E. & Wiria F. E. Analysis of the compressive behaviour of the three-dimensional printed porous titanium for dental implants using a modified cellular solid model. Proc Inst Mech Eng H. 227(9), 1020–6 (2013). 23804952 10.1177/0954411913489802

[b24] Jing D. . Effect of low-level mechanical vibration on osteogenesis and osseointegration of porous titanium implants in the repair of long bone defects. Sci Rep. 5, 17134, doi: 10.1038/srep17134 (2015). 26601709 PMC4658533

[b25] Ahmad Agha N., Willumeit-Römer R., Laipple D., Luthringer B. & Feyerabend F. The Degradation Interface of Magnesium Based Alloys in Direct Contact with Human Primary Osteoblast Cells. PLoS One. 11(6), e0157874, doi: 10.1371/journal.pone.0157874. eCollection (2016). 27327435 PMC4915630

[b26] Wang J., Xu J., Liu W., Li Y. & Qin L. Biodegradable Magnesium (Mg) Implantation Does Not Impose Related Metabolic Disorders in Rats with Chronic Renal Failure. Sci Rep. 6, 26341, doi: 10.1038/srep26341 (2016). 27210744 PMC4876325

[b27] Fariñas J. C., Rucandio I., Pomares-Alfonso M. S., Villanueva-Tagle M. E. & Larrea M. T. Determination of rare earth and concomitant elements in magnesium alloys by inductively coupled plasma optical emission spectrometry. Talanta. 154, 53–62 (2016). 27154648 10.1016/j.talanta.2016.03.047

[b28] LentzM., RisseM., SchaeferN., ReimersW. & BeyerleinI. J. Strength and ductility with {10 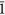 1}-{10 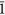 2} double twinning in a magnesium alloy. Nat Commun. 7, 11068, doi: 10.1038/ncomms11068 (2016).27040648PMC5482718

[b29] Park K. D., Jung Y. S., Lee K. K. & Park H. J. Behavior of Osteoblast-Like Cells on a β-Tricalcium Phosphate Synthetic Scaffold Coated With Calcium Phosphate and Magnesium. J Craniofac Surg. 27(4), 898–903 (2016). 27244203 10.1097/SCS.0000000000002651

[b30] Ding W. Opportunities and challenges for the biodegradable magnesium alloys as next-generation biomaterials. Regen Biomater. 3(2), 79–86 (2016). 27047673 10.1093/rb/rbw003 PMC4817317

[b31] Witte F. The history of biodegradable magnesium implants: A review. Acta Biomater. 6(5), 1680–92 (2015). 10.1016/j.actbio.2010.02.028 20172057

[b32] Wang J. . Recommendation for modifying current cytotoxicity testing standards for biodegradable magnesium-based materials. Acta Biomater. 21, 237–49 (2015). 25890098 10.1016/j.actbio.2015.04.011

[b33] Zhai Z. . The effect of metallic magnesium degradation products on osteoclast-induced osteolysis and attenuation of NF-κB and NFATc1 signaling. Biomaterials. 35(24), 6299–310 (2014). 24816285 10.1016/j.biomaterials.2014.04.044

[b34] Xie Y. & Yang L. Calcium and Magnesium Ions Are Membrane-Active against Stationary-Phase Staphylococcus aureus with High Specificity. Sci Rep. 6, 20628, doi: 10.1038/srep20628 (2016). 26865182 PMC4749956

[b35] Tian J. . Investigation of the antimicrobial activity and biocompatibility of magnesium alloy coated with HA and antimicrobial peptide. J Mater Sci Mater Med. 26(2), 66, doi: 10.1007/s10856-015-5389-3. Epub 2015 (2015). 25631264

[b36] Ling Ren., Xiao Lin., Lili Tan. & Ke Yang. Effect of surface coating on antibacterial behavior of magnesium based metals, Materials Letters. 65, 3509–3511 (2011).

[b37] Zhao Y., Wang X., Xiao J., Yu B. & Li F. Ti–Cu–N hard nanocomposite films prepared by pulse biased arc ion plating. Applied Surface Science. 258, 370–376 (2011).

[b38] Zhao Y. . Ti/TiN multilayer thin films deposited by pulse biased arc ion plating. Applied Surface Science. 257, 2683–2688 (2011).

[b39] Huang Q. . Ginsenoside-Rb2 displays anti-osteoporosis effects through reducing oxidative damage and bone-resorbing cytokines during osteogenesis. Bone. 66, 306–14 (2014). 24933344 10.1016/j.bone.2014.06.010

[b40] Li Y. . Improving osteointegration and osteogenesis of three-dimensional porous Ti6Al4V scaffolds by polydopamine-assisted biomimetic hydroxyapatite coating. ACS Appl Mater Interfaces. 7(10), 5715–24 (2015). 25711714 10.1021/acsami.5b00331

[b41] Gao P. . Beta-tricalcium phosphate granules improve osteogenesis *in vitro* and establish innovative osteo-regenerators for bone tissue engineering *in vivo*. Sci Rep. 6, 23367, doi: 10.1038/srep23367 (2016). 27000963 PMC4802206

[b42] Miranda C. R., Azevedo A. F., Baldan M. R., Beloto A. F. & Ferreira N. G. A novel procedure to obtain nanocrystalline diamond/porous silicon composite by chemical vapor deposition/infiltration processes. J Nanosci Nanotechnol. 9(6), 3877–82 (2009). 19504935 10.1166/jnn.2009.ns83

[b43] Carlson S. W., Goetz D. D., Liu S. S., Greiner J. J. & Callaghan J. J. Minimum 10-Year Follow-Up of Cementless Total Hip Arthroplasty Using a Contemporary Triple-Tapered Titanium Stem. J Arthroplasty. 31(10), 2231–6 (2016). 27339824 10.1016/j.arth.2016.04.037

[b44] Gornet M. F., Singh V., Schranck F. W., Skipor A. K. & Jacobs J. J. Serum Metal Concentrations in Subjects with Titanium Ceramic Composite Cervical Disc Replacements. Spine (Phila Pa 1976) [Epub ahead of print] (2016). 10.1097/BRS.0000000000001745 27323223

[b45] Özcan M. & Volpato C. Â. Adhesion Protocol for Bonding Abutments or Fixed Dental Prostheses on Titanium Bases in Implant-borne Reconstructions: How and Why? J Adhes Dent. 18(3), 268–9 (2016). 27341387 10.3290/j.jad.a36449

[b46] Tunchel S., Blay A., Kolerman R., Mijiritsky E. & Shibli J. A. 3D Printing/Additive Manufacturing Single Titanium Dental Implants: A Prospective Multicenter Study with 3 Years of Follow-Up. Int J Dent. 2016, 8590971, doi: 10.1155/2016/8590971 (2016). 27313616 PMC4903129

[b47] Sirolli M., Mafra C. E., Santos R. A., Holzhausen L. S., César J. B. & Neto. Influence of Piezosurgery on Bone Healing around Titanium Implants: A Histological Study in Rats. Braz Dent J. 27(3), 278–283 (2016). 27224560 10.1590/0103-6440201600161

[b48] Walker J., Shadanbaz S., Woodfield T. B., Staiger M. P. & Dias G. J. Magnesium biomaterials for orthopedic application: a review from a biological perspective. J Biomed Mater Res B Appl Biomater. 102(6), 1316–31 (2014). 24458999 10.1002/jbm.b.33113

[b49] Ostrowski N. J., Lee B., Roy A., Ramanathan M. & Kumta P. N. Biodegradable poly(lactide-co-glycolide) coatings on magnesium alloys for orthopedic applications. J Mater Sci Mater Med. 24(1), 85–96 (2013). 23053803 10.1007/s10856-012-4773-5

[b50] Chen Y., Xu Z., Smith C. & Sankar J. Recent advances on the development of magnesium alloys for biodegradable implants. Acta Biomater. 10(11), 4561–73 (2014). 25034646 10.1016/j.actbio.2014.07.005

[b51] Kirkland N. T., Birbilis N. & Staiger M. P. Assessing the corrosion of biodegradable magnesium implants: a critical review of current methodologies and their limitations. Acta Biomater. 8(3), 925–36 (2012). 22134164 10.1016/j.actbio.2011.11.014

[b52] Staiger M. P., Pietak A. M., Huadmai J. & Dias G. Magnesium and its alloys as orthopedic biomaterials: a review. Biomaterials. 27(9), 1728–34 (2006). 16246414 10.1016/j.biomaterials.2005.10.003

[b53] Saleh M. M., Touny A. H., Al-Omair M. A. & Saleh M. M. Biodegradable/biocompatible coated metal implants for orthopedic applications. Biomed Mater Eng. 27(1), 87–99 (2016). 27175470 10.3233/BME-161568

[b54] Li X. . Design of magnesium alloys with controllable degradation for biomedical implants: From bulk to surface. Acta Biomater. 45, 2–30 (2016). 27612959 10.1016/j.actbio.2016.09.005

[b55] Zhang Y. . Implant-derived magnesium induces local neuronal production of CGRP to improve bone-fracture healing in rats. Nat Med. 22(10), 1160–1169 (2016). 27571347 10.1038/nm.4162 PMC5293535

